# Phenolic Profiling for Traceability of *Vanilla* ×*tahitensis*

**DOI:** 10.3389/fpls.2017.01746

**Published:** 2017-10-12

**Authors:** Matteo Busconi, Luigi Lucini, Giovanna Soffritti, Jamila Bernardi, Letizia Bernardo, Christel Brunschwig, Sandra Lepers-Andrzejewski, Phila Raharivelomanana, Jose A. Fernandez

**Affiliations:** ^1^Department of Sustainable Crop Production, Università Cattolica del Sacro Cuore, Piacenza, Italy; ^2^Institute of Environmental and Agricultural Chemistry, Università Cattolica del Sacro Cuore, Piacenza, Italy; ^3^Equipe EIMS (Etude Intégrée des Métabolites Secondaires), UMR 241 EIO Université de la Polynésie Française, Tahiti, French Polynesia; ^4^Département Recherche et Développement, Etablissement Vanille de Tahiti, Raiatea, French Polynesia; ^5^IDR- Laboratorio de Biotecnología y Recursos Naturales, Universidad de Castilla-La Mancha, Albacete, Spain

**Keywords:** *Vanilla* ×*tahitensis*, food metabolomics, phenolics, traceability, authenticity

## Abstract

Vanilla is a flavoring recovered from the cured beans of the orchid genus *Vanilla*. *Vanilla* ×*tahitensis* is traditionally cultivated on the islands of French Polynesia, where vanilla vines were first introduced during the nineteenth century and, since the 1960s, have been introduced to other Pacific countries such as Papua New Guinea (PNG), cultivated and sold as “Tahitian vanilla,” although both sensory properties and aspect are different. From an economic point of view, it is important to ensure *V*. ×*tahitensis* traceability and to guarantee that the marketed product is part of the future protected designation of the origin “Tahitian vanilla” (PDO), currently in progress in French Polynesia. The application of metabolomics, allowing the detection and simultaneous analysis of hundreds or thousands of metabolites from different matrices, has recently gained high interest in food traceability. Here, metabolomics analysis of phenolic compounds profiles was successfully applied for the first time to *V*. ×*tahitensis* to deepen our knowledge of vanilla metabolome, focusing on phenolics compounds, for traceability purposes. Phenolics were screened through a quadrupole-time-of-flight mass spectrometer coupled to a UHPLC liquid chromatography system, and 260 different compounds were clearly evidenced and subjected to different statistical analysis in order to enable the discrimination of the samples based on their origin. Eighty-eight and twenty three compounds, with a prevalence of flavonoids, resulted to be highly discriminant through ANOVA and Orthogonal Projections to Latent Structures Discriminant Analysis (OPLS-DA) respectively. Volcano plot analysis and pairwise comparisons were carried out to determine those compounds, mainly responsible for the differences among samples as a consequence of either origin or cultivar. The samples from PNG were clearly different from the Tahitian samples that were further divided in two different groups based on the different phenolic patterns. Among the 260 compounds, metabolomics analysis enabled the detection of previously unreported phenolics in vanilla (such as flavonoids, lignans, stilbenes and other polyphenols).

## Introduction

Vanilla is a flavoring traditionally recovered from the cured beans of the orchid genus *Vanilla*. It is also one of the three most expensive spices in the world, along with saffron and cardamom (Hondrogiannis et al., 2013). Other than as a fragrance, in traditional Mexican medicine vanilla was considered as a medicinal plant with multiple positive effects on men's health (Rain and Lubinsky, 2011). In eighteenth and nineteenth centuries, vanilla was included in the European and American pharmacopeia for its medicinal uses (King et al., 1898; Bythrow, 2005). Anti-inflammatory, antiviral, analgesic, antiseptic and aesthetic properties of vanilla have been recently reported (Duke et al., 2003). Antioxidant properties of vanilla and vanilla constituents (essential oil or extract) have been reported by different authors (Kumar et al., 2002; Teuscher et al., 2005; Maurya et al., 2007). The genus *Vanilla* is indigenous of Central America, and in particular of Mexico, and comprises over 100 different species (Soto Arenas, 2003; Soto Arenas and Dressler, 2010), of which only two are currently cultivated for commercial purposes, *Vanilla planifolia* Jacks. ex Andrews and *Vanilla* ×*tahitensis* (previously *V. tahitensis* Moore). Vanilla plants are grown in hot-humid tropical climates and have certain agro-ecological requirements in terms of temperature (20–32°C), precipitation (average from 2,000 to 3,000 mm per year), altitude (from sea level to 600 m), shade (50–70%), well-drained soil rich in humus, support trees (as they are hemi-epiphytic orchids). Optimal flowering, and, consequently, pod production, requires specific climatic conditions of a dry and cool season of at least 2 months (Hernandez and Lubinsky, 2011). The hybrid nature of Tahitian vanilla was recently determined (Lubinsky et al., 2008). Analysis of cpDNA and nuclear ITS sequences provided evidence that Tahitian vanilla is a hybrid between *V. planifolia* and *V. odorata* C. Presl, with *V. planifolia* being the female parent. *Vanilla* ×*tahitensis* is traditionally cultivated on French Polynesian islands, where vanilla vines were first introduced during the nineteenth century (Costantin and Bois, 1915; Bouriquet, 1954; Florence and Guérin, 1996; Lepers-Andrzejewski et al., 2012), currently, vanilla production is mainly carried out in the Leeward Islands (high islands: Raiatea, Tahaa, Huahine), Society archipelago (Lepers-Andrzejewski and Dron, 2010). Subsequently, within a short period of time, diversification resulted in the origination of about 14 cultivars identified over the years by local producers (Lepers-Andrzejewski et al., 2010). Among the isolated cultivars, two of them, “Haapape” and “Tahiti,” which are morphologically and genetically differentiated (Lepers-Andrzejewski et al., 2011), became the most widespread and most commercialized cultivars in French Polynesia. Since the 1960s, *V*. ×*tahitensis* has been introduced into other Pacific countries such as Papua New Guinea (PNG), cultivated and sold as “Tahitian vanilla,” although both sensory properties and aspect are different. These differences depend more on factors such as genetic traits, environment and technology (curing method and storage conditions). Actually, *V*. ×*tahitensis*, being less restrictive to humidity and supporting better rainfall, was found to be more suitable to the climate of PNG than *V. planifolia* and the Sepik area (well-drained alluvial plains) is the main vanilla production region (Mac Gregor, 2005). The volatile composition of vanilla in general and of *V*. ×*tahitensis* in particular has recently been investigated and the results were reported in the literature (Pérez-Silva et al., 2006; Brunschwig et al., 2009; Lepers-Andrzejewski et al., 2010; Brunschwig et al., 2012; Takahashi et al., 2013; Brunschwig et al., 2016).

According to official data (http://www.fao.org/faostat), in 2014, global vanilla production was over 7,000 t, with French Polynesia ranking ninth among the vanilla producers in the world. Considering the high economic value of vanilla, in the last years, several publications have dealt with the development of reliable methods to trace vanilla production according to the species or the different geographic origin. Tracing the genetic and/or the geographic origin of vanilla is crucial because the species, the variety, the environment (in particular climatic conditions) and the production method (curing methods and storage) imply characteristic different flavors that could be reflected in the quality and the price of the product. From an economic point of view, it is important to ensure *V*. ×*tahitensis* traceability and to guarantee that the product which is marketed is part of the future protected designation of the origin “Tahitian vanilla” (PDO) (Journal Officiel de la Polynésie Française, 2014, 2016). *Vanilla* ×*tahitensis* traceability has already been carried out via different analytical techniques, such as gas chromatography–flame ionization detection (GC-FID) and gas chromatography–mass spectrometry (GC-MS), to analyse the volatile compounds for quality control (Brunschwig et al., 2016); analysis of the stable isotopes of carbon and hydrogen evidencing that *V*. ×*tahitensis* has more heavy carbon than *V. planifolia* and that isotopes can be used to discriminate the geographic origin of the samples (Sølvbjerg Hansen et al., 2014); wavelength dispersive X-ray fluorescence to identify the elemental composition and the geographic origin of vanilla samples (Hondrogiannis et al., 2013). The use of metabolomics, allowing the detection and simultaneous analysis of hundreds or thousands of metabolites from different matrices, has recently gained high interest in food traceability (Oms-Oliu et al., 2013). Metabolomic techniques have been applied for the analysis of raw food material (cultivar identification, study of different metabolites that accumulate during plant growth, ripening and postharvest) and processed plant-derived food (food classification, authenticity assessment, food control) (Oms-Oliu et al., 2013). Up to now, the study of vanilla metabolome was applied to *V. planifolia*, albeit not for traceability purposes. Some studies concerning the metabolome of *V. planifolia* green pods from La Réunion (Palama et al., 2011) and the changes in metabolome composition in leaves (Palama et al., 2010) and pods (Palama et al., 2009) in different developmental stages have recently been published. Recently, Gu et al. (2017) carried out a comparative metabolomics analysis by using high-performance liquid chromatography–mass spectrometry (LC–MS) to analyse vanilla metabolome before and after curing to study the biosynthesis of vanillin during the curing process of vanilla. They evidenced the presence of at least seven different putative pathways of vanillin biosynthesis some of them possibly correlated with microbial activity.

In other species, metabolomics analysis was applied successfully to assess, among others, the authenticity of processed plant-derived food such as fruit juices (Vardin et al., 2008), coffee (Oliveira et al., 2009), vegetable oils (Rohman and Man, 2012; Ruiz-Samblás et al., 2012), and tomatoes (Lucini et al., 2017).

In the present work, we applied, for the first time, the comprehensive profile of the phenolic compounds for traceability purposes in *V*. ×*tahitensis*. Samples from two different areas, French Polynesia (FP) and PNG, were surveyed and the profile of the phenolic compounds investigated by UHPLC-ESI/QTOF-MS.

## Materials and methods

### Vanilla samples

Two commercial samples of *Vanilla* ×*tahitensis* pods were obtained from two commercial providers (“Pacific Natural Product” and “Tahiti vanille” Brand); both of these samples were grown on the Leeward Islands and harvested in 2013. The pods have been cured following the traditional Polynesian method (Lepers-Andrzejewski et al., 2010). A sample of *V*. ×*tahitensis* pods (250 g) from PNG was provided by the NARI organization (PNG's National Agricultural Research Institute, donation from Dr. Sergie Bang) from the East Sepik region and collected from the 2013 harvest. It was used as reference for a comparison with *V*. ×*tahitensis* from French Polynesia. The pods have been cured according to the methods currently used in PNG (see discussion). At the end, three batches were available: batch1, pods of Tahitian vanilla, belonging to the “Haapape” cultivar, Vanille de Tahiti Brand from Pacific Natural Product, grown on the Leeward Islands (French Polynesia FP); batch2, mixture of pods of Tahitian vanilla, cultivars “Haapape” and “Tahiti,” “Tahiti vanille” Brand, from the Leeward Islands (FP); batch3, pods of *V*.×*tahitensis* from PNG.

### Profiling of phenolic compounds

Ten independent pods per treatment were analyzed as individual samples. The phenolic compounds were screened through a quadrupole-time-of-flight mass spectrometer coupled to a UHPLC liquid chromatography system (UHPLC-ESI/QTOF-MS), on the basis of the approach described by Lucini et al. (2015). Samples were extracted in 10 volumes of 50 mM HCOOH in 80% methanol, using an IKA T10 Ultra-Turrax to comminute samples (3 min at 30,000 rpm). The extracts were then centrifuged at +4°C and filtered through a 0.22-μm cellulose membrane, diluted five times in 50% methanol and transferred to an amber vial for LC-ESI/Q-TOF-MS analysis. A 1290 UHPLC liquid chromatograph, equipped with a binary pump and coupled to a G6550 iFunnel QTOF mass spectrometer through a Dual Electrospray JetStream ionization system (all from Agilent Technologies Santa Clara, CA, USA), was used to profile phenolic compounds. The mass spectrometer was operated in positive MS-only (SCAN) mode to acquire spectra in the range 50–1,000 m/z. Extracts were injected (6 μL) and chromatographed under a water-methanol gradient elution (from 6% methanol to 92% methanol in 35 min), using an Agilent Zorbax Eclipse Plus C18 column (50 × 2.1 mm, 1.8 μm). Lock masses and source conditions were optimized for phenolic compounds in previous experiments (Lucini et al., 2017). Briefly, nitrogen was used as drying gas (8 L min^−1^ and 330°C), nebulizer pressure was 60 psig and capillary voltage was 3,500 V. Blanks were analyzed between samples and lock masses (m/z 121.0509 and 922.0098) were continuously infused during chromatographic runs to achieve higher accuracies.

Raw data were processed via the Agilent Profinder B.0700 software using the “find-by-formula” algorithm. With this purpose, features mass and retention time were aligned and then the whole isotopic profile (isotopic spacing and isotopic ratio) was used for compounds' annotation, together with the monoisotopic accurate mass, against the database exported from Phenol-Explorer 3.6 (Rothwell et al., 2013). In addition, recursive analysis (using retention time as mandatory in the second ID step, with a tolerance of 0.1 min) and frequency filter were applied (only those compounds being in at least 80% of replications within at least one condition were retained). Therefore, based on the strategy applied, identification was carried out according to Level 2 (putatively annotated compounds) as set out by the COSMOS Metabolomics Standards Initiative (http://cosmos-fp7.eu/msi).

### Statistical analysis

The abundance value for each compound in the dataset was log2-transformed, normalized at 75th percentile and baselined to the median in the dataset. One-way analysis of variance (ANOVA) (*p* < 0.05, Benjamini-Hochberg multiple testing correction) has been carried out on the starting data set of metabolites. Unpaired *t*-test (*p* < 0.05, Benjamini-Hochberg FDR multiple testing correction) and fold-change analysis (cut-off = 2) were combined into Volcano plot analysis. Subsequently, unsupervised hierarchical cluster analysis (Euclidean similarity measure and Wards linkage rule) was generated on the basis of fold-change heatmaps.

Finally, the raw dataset was exported in SIMCA 14 (Umetrics, Malmo, Sweden), Pareto scaled (to reduce the relative importance of larger values and partially preserve data structure) and elaborated for orthogonal partial least squares discriminant analysis (OPLS-DA) prediction modeling. Herein, the variation between the three batches was separated into predictive and orthogonal (technical and biological variation) components. The presence of outliers was investigated according to Hotelling's T2 (i.e., the distance from the origin in the model plane), using 95 and 99% confidence limits for suspect and strong outliers, respectively. Method validity was next tested using CV-ANOVA (*P* < 0.01) and permutation testing after inspecting model parameters (goodness-of-fit R^2^Y and goodness-of-prediction Q^2^Y). Regarding Q^2^Y prediction ability, a value > 0.5 indicates good model quality (Rombouts et al., 2017). Variable importance in projection (VIP analysis) was used to evaluate the importance of metabolites and select the most discriminant ones (VIP score > 1).

## Results

**Figure 1:**
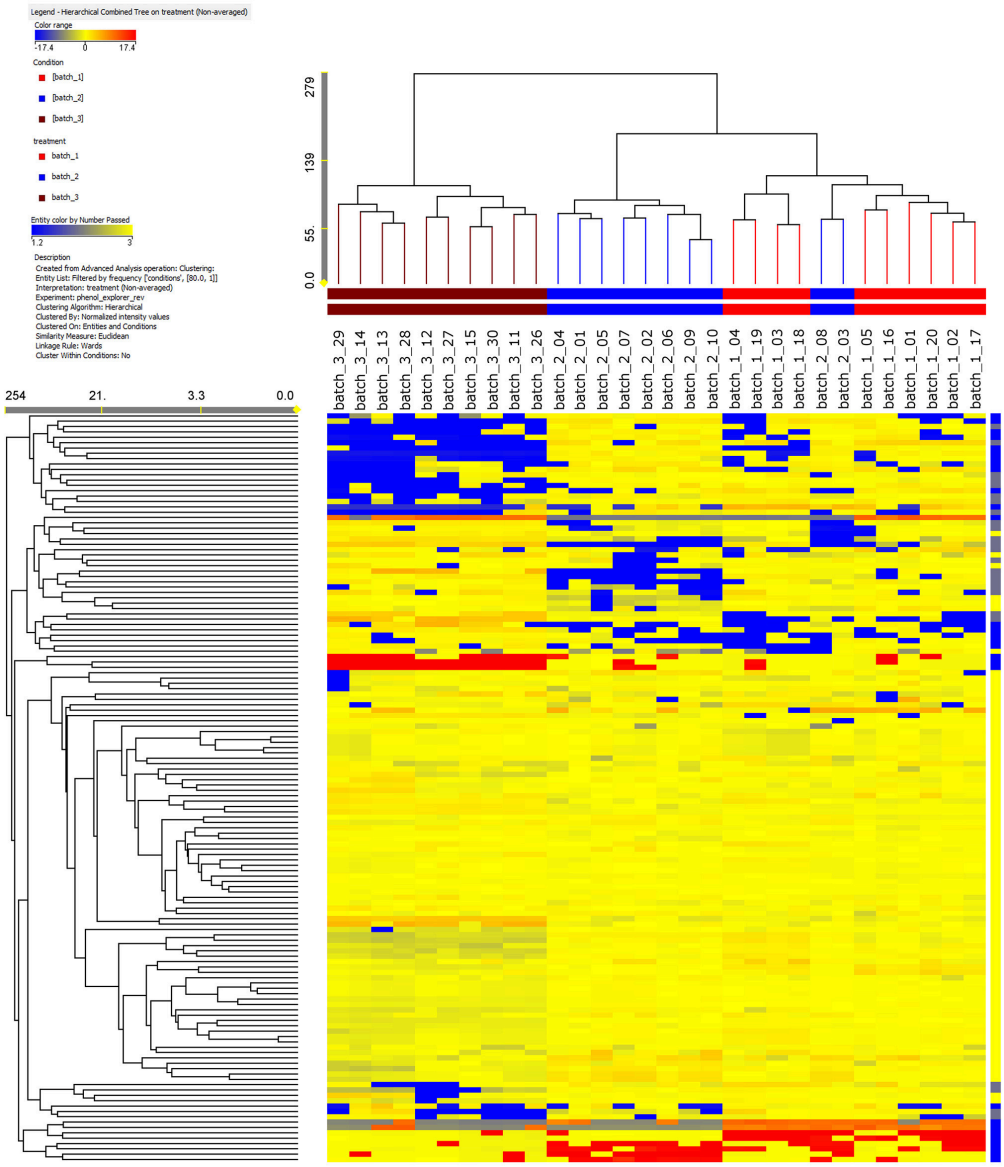
Results of a not averaged, unsupervised hierarchical cluster analysis on the phenolic profile in the vanilla pods analyzed. The intensity of the compounds was used to build the heat maps on which the clusters were generated. Samples from PNG (brown) are clearly different with respect to Tahitian samples (red, batch1, and blue, batch2) in regards to the phenolic composition. Among the Tahitian samples, two patterns can be distinguished.

Ten single cured pods were recovered from each of the three vanilla batches and analyzed independently from the others. The use of an informative approach, such as UHPLC-ESI/QTOF-MS together with a comprehensive database (Phenol-Explorer), allowed the annotation of 260 phenolic compounds. All these phenolics belonged to a small number of main classes: flavonoids (120 compounds), phenolic acids (53 compounds), lignans (18 compounds), stilbenes (6 compounds) and one last group labeled as “other polyphenols” (63 compounds) (Supplementary Table 1). To the best of our knowledge, we report for the first time the occurrence of phenolic compounds from *V*. ×*tahitensis*, which do not belong to flavoring components (mainly composed by volatile components), such as flavonoids, lignans, stilbenes and other classes of polyphenols (curcuminoids). Based on the fold-change analysis, a heat map was developed and subsequently, an unsupervised hierarchical cluster analysis was carried out (Figure 1). It is important to underline that with the applied method we did not obtain an absolute quantification of the compounds but an indication of the relative abundance, based on the area of the peaks, of the different phenolics in the samples under comparison. Three main clusters could be identified: (1) all the pods from PNG batch3 (brown cluster); (2) eight pods from FP batch2 (blue cluster); (3) all the pods from FP batch1 (red cluster) plus two pods from FP batch2. Samples from PNG presented a characteristic phenolic profile, differing from that of the Tahitian ones, thus indicating that samples could be discriminated in terms of their geographic origin. Within the Tahitian pods, the metabolic profiles were quite similar, but still sufficiently different to allow two further sub-clusters to separate the Tahitian sample sets.

Two approaches were then applied to investigate the most differential compounds within the sample set. Initially, the data set was analyzed by using a one-way ANOVA (*P* < 0.05) that resulted in 88 differential compounds out of the 260 phenolics identified (Supplementary Table 2). Flavonoids were the most represented compounds (39 compounds), followed by “other polyphenols” and phenolic acids (21 and 20 compounds, respectively), stilbenes and lignans (four compounds per class). Among the phenolic acids, hydroxycinnamic acids were the most represented subclass (13 compounds) while within the flavonoids, flavonols (nine compounds), flavones (eight compounds) and anthocyanins (eight compounds) were more represented. Vanillin (4-Hydroxy-3-methoxybenzaldehyde) content was significantly different between the three samples according to one-way ANOVA, but the fold change was not high enough to be recognized as discriminant with the Volcano plot analysis in the pairwise comparison (see later). Among the 260 compounds, only one anisyl derivative (anisaldehyde) was detected, but not selected among the most discriminant compounds with ANOVA nor with the subsequent analyses (OPLS-DA and Volcano plot). This highlights that such metabolomics analysis was more powerful to detect, in *V*. ×*tahitensis*, compounds even more discriminant than the characteristic odor-active anisyl compounds (Brunschwig et al., 2012, 2016).

**Figure 2:**
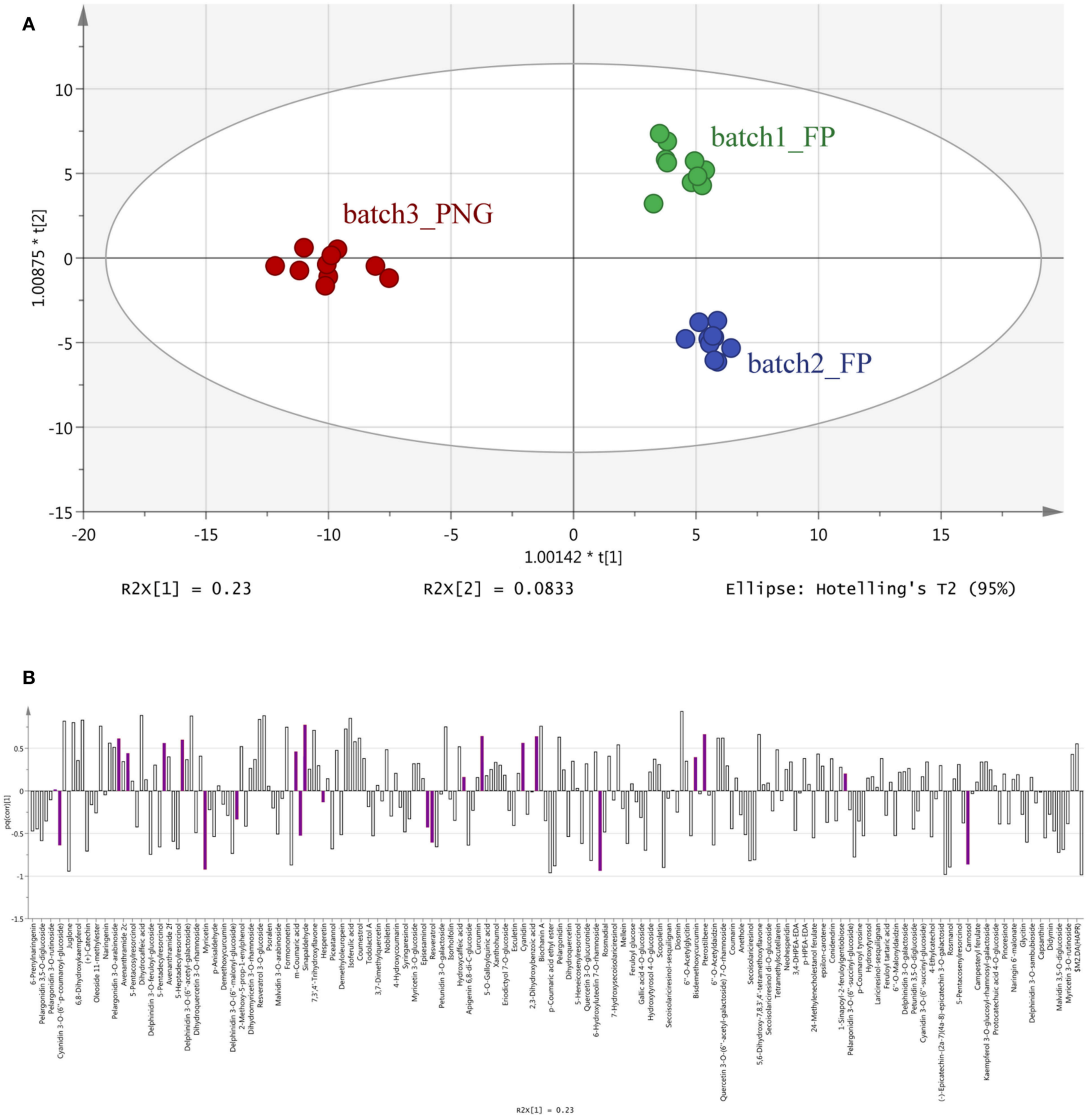
Orthogonal Projections to Latent Structures Discriminant Analysis (OPLS-DA) on vanilla sample phenolic profiles. Individual replications are given in the model score plot **(A)**, whereas loadings column plot is reported in the **(B)**. Compounds selected by VIP analysis are reported in color in the loadings column plot. PNG samples (red samples) are clustered on the left whereas Tahitian samples (green and blue samples) on the right side of the plot. Both geographic origin and cultivars were responsible for the actual phenolic signature of the samples.

The results of OPLS-DA agreed with the unsupervised cluster analysis being able to separate the samples according to their origin and, possibly, the cultivar of origin (Figure 2). Indeed, the characteristics of the model were excellent: R^2^Y = 0.969 and Q^2^Y = 0.877. No outlier samples could be observed by Hotelling's T2, whereas both CV-ANOVA (*P* = 4.3 10^−14^ for regression) and permutation test (Supplementary Figure 2) showed a more than adequate degree of validation.

Overall, all these results evidenced that differences among the three samples were included in the compounds data set, thus driving the need for a more detailed assessment of which compounds these differences could be ascribed too. The VIP analysis from OPLS-DA allowed to select a small number of discriminant compounds, reducing the data set to only 23 highly discriminant compounds. Individual VIP scores are reported in Table 1, together with the discriminant compounds grouped in phenolic classes. Flavonoids (12 different compounds), other polyphenols and phenolic acids (four different compounds each one) were the most represented subclasses of phenolics (Table 1). Among the most discriminant compounds, two stilbenes (resveratrol and pterostilbene) have been detected.

**Table 1:** Compounds discriminating between the three vanilla samples, as obtained from VIP analysis from Orthogonal Projections to Latent Structures Discriminant Analysis (OPLS-DA).

**Class**	**Sub class**	**Compound**	**VIP score**
Flavonoids	Anthocyanins	Cyanidin 3-*O*-(6″-*p*-coumaroyl-glucoside)	1.2655
		Cyanidin	1.5515
		Peonidin	1.6544
		Kaempferol	1.5474
			
	Flavanols	(-)-Epigallocatechin	1.1652
	Flavanones	6-Geranylnaringenin	1.6041
		Hesperetin	1.2650
		Naringenin 7-O-glucoside	1.5636
	Flavones	Cirsilineol	1.5881
		7,4′-Dihydroxyflavone	1.6009
	Flavonols	Myricetin	1.4220
		Kaempferide	1.6410
Lignans	Lignans	Dimethylmatairesinol	1.4705
Other polyphenols	Curcuminoids	Bisdemethoxycurcumin	1.6693
	Hydroxybenzoketones	2,3-Dihydroxy-1-guaiacylpropanone	1.0357
		3-Methoxyacetophenone	1.6664
	Phenolic terpenes	Carnosol	1.3296
Phenolic acids	Hydroxycinnamic acids	*m*-Coumaric acid	1.7628
		Sinapic acid	1.3886
	Hydroxycinnamaldehydes	Sinapaldehyde	1.5979
	Hydroxyphenylpropanoic acids	3,4-dihydroxyphenyl-2-oxypropanoic acid	1.4280
Stilbenes	Stilbenes	Resveratrol	1.4601
		Pterostilbene	1.4923

Volcano plot analysis (unpaired *t*-test; *p* ≤ 0.01; fold-change cut off = 2) was carried out to determine those compounds mainly responsible for the differences among samples as a consequence of either origin or cultivar. Three different pairwise comparisons were carried out: (1) between the two Tahitian batch samples; (2) between FP batch2 and PNG; (3) between FP batch1 and PNG. The discriminant compounds for all the three different comparisons have been reported in Supplementary Table 3, in three active sheets, along with the corresponding fold change (an estimate of the relative abundance of the compounds in the samples under pairwise comparison) and regulation. The volcano plot graphic output of the comparisons FP batch1 and PNG and FP batch2 and PNG is reported in Supplementary Figure 1.

Twenty-one compounds (Table 2) were discriminant between the samples from the same geographic location (French Polynesia): flavonoids (10 compounds, highly represented by flavonols) and phenolic acids (five compounds, almost exclusively hydroxycinnamic acids) were the most frequent classes of phenolics. Discriminant hydroxycinnamic acids were derivatives of coumaric and caffeic acid. Six compounds were grouped under a class named, in phenol-explorer (Rothwell et al., 2013), as “other polyphenols.” Twelve compounds were up-regulated, while nine were down-regulated in FP batch1 with respect to the mixture (batch2). Fifty percent of flavonoids were respectively up- and down-regulated. Among other classes of phenolics, the up-regulated compounds in FP batch1 prevailed. Those compounds identified as discriminant through both the *t*-test and OPLS-DA are marked in Table 2 with an asterisk.

**Table 2:** Discriminant metabolites differentiating pods of batch1 from those of batch2.

**Class**	**Sub class**	**Compound**	**Regulation**	***p*-Value**
Flavonoids	Flavones	Cirsilineol ^*^	Down	2.65E-07
	Flavanones	6-Geranylnaringenin ^*^	Up	0.002808
	Flavonols	Kaempferide ^*^	Down	9.43E-08
		3,7-Dimethylquercetin	Down	6.97E-06
		Quercetin 3-O-acetyl-rhamnoside	Down	4.68E-04
		Myricetin ^*^	Up	5.46E-03
		Isorhamnetin 3-O-glucuronide	Up	1.06E-05
		(-)-Epigallocatechin ^*^	Up	1.59E-03
	Dihydroflavonols	Dihydroquercetin 3-O-rhamnoside	Up	0.007018
	Anthocyanins	Peonidin ^*^	Down	1.99E-08
Phenolic acids	Hydroxycinnamic acids	Hydroxycaffeic acid	Down	0.003478
		m-Coumaric acid ^*^	Up	3.63E-08
		Caffeoyl tartaric acid	Up	6.63E-03
		p-Coumaroyl tartaric acid	Up	1.19E-05
	Hydroxyphenylpropanoic acids	3,4-dihydroxyphenyl-2-oxypropanoic acid ^*^	Up	1.82E-05
Other polyphenols	Alkylphenols	5-Heneicosylresorcinol	Down	6.88E-03
		5-Pentadecylresorcinol	Up	2.05E-03
	Hydroxybenzoketones	2,3-Dihydroxy-1-guaiacylpropanone ^*^	Down	6.45E-06
	Phenolic terpenes	Carnosol ^*^	Down	5.74E-04
	Hydroxycoumarins	Esculin	Up	2.81E-04
	Curcuminoids	Bisdemethoxycurcumin ^*^	Up	7.42E-06

*The compounds were grouped in their chemical classes and sub-classes. The up- and down-regulation and the corresponding p values, as a result of the Volcano analysis (p ≤ 0.05, fold-change cut-off = 2.0), are reported*.

* *Compounds also recognized as discriminant via OPLS-DA analysis*.

Contrary to what was observed between the Tahitian samples, the other comparisons evidenced a higher number of differential compounds, respectively, 82 (FP batch2 vs. PNG) and 57 (FP batch1 vs. PNG). Checking these differential compounds through Venn analysis (Supplementary Figure 1), 46 metabolites were found in both the comparisons, while 36 and 11, respectively, were exclusive of the single comparisons for the two Tahitian batches when compared to PNG (Tables 3, 4). The common metabolites showed always the same kind of regulation in Tahitian samples, up or down, respectively, with respect to PNG samples; no compounds showing different regulations in the two comparisons could be observed (Table 3). The *p*-values of the common metabolites, as resulted from the two different pairwise comparisons, have been reported in Table 3. As an example, focusing on stilbenes, pterostilbene and resveratrol 3-*O*-glucoside were highly up-regulated, while piceatannol was down-regulated, both in batch2 vs. PNG and in batch1 vs. PNG. Among these compounds responsible for discrimination between the two different locations, flavonoids were the most represented ones (20 compounds), followed by phenolic acids (nine compounds). Within flavonoids, anthocyanins and flavonols were the most abundant compounds and glycosylated forms (e.g., malvidin 3-*O*-galactoside, petunidin 3-*O*-galactoside, petunidin 3-*O*-rutinoside) were highly represented. Globally, 31 and 15 compounds, respectively, were significantly up- and down-regulated in Tahitian samples. In more detail, flavonoids (17 out of 20) and phenolic acids (6 out of 9) were mainly up-regulated, while within other polyphenols, more or less the same number of compounds were up- or down-regulated (5 and 6 out of 11, respectively). Eight of the compounds reported in Table 3 were also selected by OPLS-DA as differential molecules able to discriminate samples based on both the geographic origin and genotype (five out of eight were up-regulated in Tahitian samples).

**Table 3:** Common metabolites, differentiating Tahitian and PNG samples, evidenced by matching the results of the two Volcano analyses: batch2 vs. PNG (b2-PNG) and batch1 vs. PNG (b1-PNG).

**Class**	**Sub class**	**Compound**	**Regulation**	***p*****-value**	
				**b2-PNG**	**b1-PNG**
Flavonoids	Flavones	7,4′-Dihydroxyflavone ^*^	Up	3.32E-05	6.72E-09
		7,3′,4′-Trihydroxyflavone	Up	1.11E-11	2.75E-07
	Flavanones	Sakuranetin	Up	0.003981	0.002101
		Eriodictyol	Up	2.41E-05	4.88E-05
	Flavonols	Myricetin ^*^	Down	3.57E-04	6.00E-04
		Isorhamnetin	Up	2.86E-07	1.51E-04
		Isorhamnetin 3-O-glucoside 7-O-rhamnoside	Up	1.49E-08	5.36E-04
		Quercetin 3-O-(6″-acetyl-galactoside) 7-O-rhamnoside	Up	1.41E-08	0.01372
	Anthocyanins	Malvidin 3-O-galactoside	Down	0.001473	0.004629
		Peonidin ^*^	Up	2.25E-08	2.34E-03
		Pelargonidin	Up	2.56E-12	1.33E-07
		Pelargonidin 3-O-arabinoside	Up	2.50E-05	1.61E-05
		Petunidin 3-O-galactoside	Up	0.006946	0.005985
		Petunidin 3-O-rutinoside	Up	2.01E-24	1.35E-04
	Dihydrochalcones	Phloretin	Down	9.83E-08	4.36E-06
		Phloretin 2′-O-xylosyl-glucoside	Up	0.012373	3.56E-04
	Isoflavonoids	Formononetin	Up	3.38E-05	6.28E-04
		6″-O-Acetylgenistin	Up	0.015696	1.19E-05
		Genistin	Up	0.001106	0.005498
		6″-O-Acetylglycitin	Up	0.003609	1.62E-04
Stilbenes	Stilbenes	Piceatannol	Down	3.67E-06	0.00864
		Pterostilbene ^*^	Up	6.17E-06	1.21E-06
		Resveratrol 3-O-glucoside	Up	3.20E-06	4.27E-06
Phenolic acids	Hydroxycinnamic acids	p-Coumaric acid ethyl ester	Down	5.97E-11	1.00E-07
		3-p-Coumaroylquinic acid	Down	5.51E-04	0.003488
		p-Coumaroyl tartaric acid	Down	5.22E-27	0.01277
		m-Coumaric acid ^*^	Up	1.68E-05	2.95E-10
		Cinnamic acid	Up	0.010233	0.002471
		3-Sinapoylquinic acid	Up	1.13E-05	1.31E-05
	Hydroxyphenylpropanoic acids	Dihydrocaffeic acid	Up	3.14E-08	6.46E-07
	Hydroxyphenylacetic acids	Homoveratric acid	Up	1.42E-09	2.94E-09
	Hydroxybenzoic acids	Ellagic acid arabinoside	Up	8.98E-10	0.001704
Other polyphenols	Other polyphenols	Phlorin	Down	1.08E-05	4.22E-06
		3,4-Dihydroxyphenylglycol	Down	0.006922	0.002482
		Coumestrol	Up	1.30E-07	6.04E-08
	Naphtoquinones	Juglone	Down	1.47E-09	5.67E-14
	Phenolic terpenes	Carnosol ^*^	Down	0.005197	5.86E-07
		Rosmanol	Down	8.77E-07	4.42E-09
	Hydroxyphenylpropenes	Acetyl eugenol	Down	1.35E-09	4.54E-07
	Hydroxycinnamaldehydes	Sinapaldehyde	Up	1.03E-09	3.54E-11
	Hydroxybenzoketones	3-Methoxyacetophenone ^*^	Up	0.020033	0.010019
	Furanocoumarins	Xanthotoxin	Up	0.001288	0.001383
	Tyrosols	p-HPEA-EDA	Up	0.003026	0.01469
Lignans	Lignans	Dimethylmatairesinol ^*^	Down	0.004767	3.32E-07
		Cyclolariciresinol	Down	0.003949	0.003149
		1-Acetoxypinoresinol	Up	0.002295	0.001418

*Metabolites are reported according to the chemical class and sub-class of the compounds. The up- and down-regulation with the corresponding p values, as a result of the Volcano analysis (p ≤ 0.05, fold-change cut-off = 2.0), are reported. Each compound reported in the Table had the same regulation (up or down) in Tahitian samples in both comparisons. The fold change of up and down regulation and other statistics have been reported in Supplementary Table 3*.

* *Compounds also recognized as discriminant via OPLS-DA analysis*.

**Table 4:** Exclusive metabolites differentiating Tahitian and PNG samples.

**Class**	**Sub class**	**Compound**	**Regulation**	***p*-value**
**FP BATCH1 vs. PNG: EXCLUSIVE COMPOUNDS**				
Flavonoids	Flavones	Apigenin 6,8-di-C-glucoside	Down	0.005157
	Flavanones	6-Geranylnaringenin ^*^	Up	1.46E-08
	Flavanols	(+)-Catechin	Down	1.23E-02
		(+)-Catechin 3-O-glucose	Up	1.08E-05
	Flavonols	Kaempferol ^*^	Up	1.00E-05
		Isorhamnetin 3-O-glucuronide	Up	5.36E-04
	Anthocyanins	Cyanidin ^*^	Up	8.94E-06
Phenolic acids	Hydroxycinnamic acids	3-Feruloylquinic acid	Up	2.81E-04
Other polyphenols	Hydroxybenzoketones	3,4-dihydroxyphenyl-2-oxypropanoic acid ^*^	Up	9.65E-05
	Curcuminoids	Bisdemethoxycurcumin ^*^	Up	1.26E-08
Lignans	Lignans	Arctigenin	Up	0.011521
**FP BATCH2 vs. PNG: EXCLUSIVE COMPOUNDS**				
Flavonoids	Flavones	Cirsimaritin	Down	0.001366
		5,6-Dihydroxy-7,8,3′,4′-tetramethoxyflavone	Up	2.21E-07
		Chrysoeriol 7-O-glucoside	Down	0.001481
		Tetramethylscutellarein	Up	0.01319
		Nobiletin	Up	1.30E-04
		6-Hydroxyluteolin 7-O-rhamnoside	Up	6.57E-06
	Flavanones	Pinocembrin	Down	7.65E-07
		Naringenin 7-O-glucoside ^*^	Down	0.015904
		Naringin 6′-malonate	Up	0.016008
	Flavanols	(-)-Epigallocatechin ^*^	Down	2.67E-08
	Flavonols	Kaempferide ^*^	Up	3.09E-09
		Isorhamnetin 3-O-galactoside	Up	0.022369
		Spinacetin 3-O-glucosyl-(1-6)-glucoside	Up	0.001063
		3,7-Dimethylquercetin	Up	0,00106
		Quercetin 3-O-acetyl-rhamnoside	Up	1.41E-08
	Anthocyanins	Malvidin 3-O-arabinoside	Down	0.001473
	Dihydroflavonols	Dihydroquercetin	Down	0.024123
	Isoflavonoids	6″-O-Malonyldaidzin	Down	0.009957
	Chalcones	Xanthohumol	Up	4.56E-04
Stilbenes	Stilbenes	Pinosylvin	Up	0.021468
		d-Viniferin	Up	0.016183
Phenolic acids	Hydroxycinnamic acids	Rosmarinic acid	Down	2.39E-04
		Caffeoyl tartaric acid	Down	2.65E-04
		Hydroxycaffeic acid	Up	1.80E-07
		Avenanthramide 2c	Up	0.012336
		Avenanthramide 2f	Up	0.010568
		Feruloyl glucose	Up	1.60E-05
	Hydroxyphenylpropanoic acids	Dihydro-p-coumaric acid	Up	3.94E-07
	Hydroxyphenylacetic acids	3,4-Dihydroxyphenylacetic acid	Up	0.001156
	Hydroxybenzoic acids	Gallic acid ethyl ester	Up	1.07E-04
Other polyphenols	Other polyphenols	Pyrogallol	Up	1.68E-04
	Alkylphenols	5-Pentadecylresorcinol	Down	5.06E-05
		5-Heneicosylresorcinol	Up	0.006515
	Hydroxycoumarins	Esculin	Down	9.51E-07
	Hydroxybenzoketones	2,3-Dihydroxy-1-guaiacylpropanone ^*^	Up	0.003059
	Alkylmethoxyphenols	4-Vinylsyringol	Up	0.013802

*By matching the differential metabolites from the two Volcano analyses, these metabolites were present only in the pods of batch1 or only in batch2. Metabolites are reported according to the chemical class and sub-class of the compounds. The up- and down-regulation and the corresponding p values, as a result of the Volcano analysis (p ≤ 0.05, fold-change cut-off = 2.0), are reported. The fold change of up and down regulation and other statistics have been reported in Supplementary Table 3*.

* *Compounds also recognized as discriminant via OPLS-DA analysis*.

Focusing on the exclusive metabolites (Table 4), the number of differential compounds was lower between FP batch1 (11) vs. PNG than between FP batch2 vs. PNG (36). Furthermore, up-regulated compounds were more abundant than the down-accumulated ones, the corresponding *p*-values are reported in Table 4. Among the exclusive compounds of batch1 and of batch2, flavonoids were predominant: 7 out of 11 for batch1 and 19 out of 36 for batch2 (flavones and flavonols were particularly abundant). Exclusive compounds of batch2 were also phenolic acids (9 out of 36) and, despite the small number, stilbenes (Table 4). Among the stilbenes, both pinosylvin and delta-viniferin were up-regulated in batch2 with respect to PNG. Overall, out of five differential stilbenes (Tables 3, 4), four were up-regulated and just one was down-regulated in Tahitian samples. Nine compounds among the exclusive metabolites were also recognized by OPLS-DA analysis; seven out of nine were up-regulated in Tahitian samples.

## Discussion

While traceability is usually most relevant when it concerns public health, the possibility to discover the origin of products, ingredients and their attributes from the farm throughout the whole food chain to the consumer is gaining more importance. Regarding plant-derived products, being able to trace the origin is extremely important. This is because the characteristics of these products are strongly influenced, other than by the genetic constitution of the cultivated varieties, by environmental conditions and by local traditional curing methods, both able to influence the final metabolic profile of the product (Brunschwig et al., 2016). Genetic bases being equal, the origin of the products, considered as the sum of environment and production technologies, implies characteristic flavors, subsequently reflected in premium prices that can represent a target for falsifications and frauds. This is particularly true in the case of *V*. ×*tahitensis*. Therefore, the development of control procedures to protect the high-quality Tahitian production is of great interest and scientifically supported by the results of recent papers (Brunschwig et al., 2009, 2016; Takahashi et al., 2013). Among them, Brunschwig et al. (2016) have analyzed the volatile composition and the sensory properties of *V*. ×*tahitensis* from different geographic origins (French Polynesia and PNG), evidencing a clear difference in the composition of these compounds between the two different geographic locations. *V*. ×*tahitensis* from PNG was clearly different from *V*. ×*tahitensis* from French Polynesia. According to the authors, these differences were mainly a consequence of the curing technology. Our study could be considered as a prosecution of Brunschwig et al. (2016), although a different analytical approach was applied, to increase the knowledge on vanilla chemical composition toward the development of a traceability procedure for this product. Along with the analysis of chemical composition, the development of traceability methods based on DNA might be a topic of great interest. On this basis, we also tried to recover DNA from the cured pods. However, the techniques employed for DNA isolation from pods, both commercial kits and customized protocols, were not efficient and no DNA, or no PCR grade DNA, could be recovered (data not shown). Although further research is required to implement DNA-based approaches, these results strongly drive the analysis of secondary metabolites for traceability purposes. In the present study, we applied for the first time metabolomics, focused on the phenolic compounds, to *V*. ×*tahitensis* traceability. Ten pods for each one of the three samples (batch1 – cultivar “Haapape” FP; batch2 – “Haapape + Tahiti” FP; batch3 – PNG) were analyzed evidencing a quali-quantitative phenolic profile involving 260 compounds. The decision to focus our attention on phenolics has been driven by the fact that: (1) these secondary metabolites are highly associated to different environmental conditions, curing methods and genotypes, thus showing high discriminant capacity (Klockmann et al., 2016); (2) such an in-depth analysis of these chemically diverse compounds was not previously carried out for this species; (3) with respect to volatiles, phenolics are expected to be more stable during storage.

Pods from FP were softer than pods from PNG and this is very likely a direct consequence of the two different curing methods adopted: in the traditional FP method, the pods are harvested when fully mature, exposed in the shade for a natural browning (no high-temperature scalding step to stop maturation), alternatively dried in the sun and wrapped in cotton material overnight; they are then finally air-dried to stabilize the flavor and keep the water content at about 50%; in the PNG method it is included a high-temperature scalding step to stop maturation and drying to about 40% water content. Using ten individual pods for each samples increases the number of independent replicates strongly supporting the reliability of the results. Flavonoids were the most abundant phenolics, followed by phenolic acids and by a high number of compounds classified as other polyphenols. Small, albeit significant was the presence of stilbenes, such as resveratrol, molecules that have recently been widely studied, mainly in grapevine, for their role in protecting plants against diseases (Bavaresco et al., 2016) and for their health benefits because of a potent antioxidant activity (Marques et al., 2009). It was noted that out of five differential stilbenes, four were highly up-regulated in Tahitian samples.

Unsupervised cluster analysis (Figure 1) clearly evidenced that the whole phenolic profile was able to separate the three batches in different clusters based on the origin: PNG vanilla was clearly different from FP vanilla. Likely, the differences between Tahitian and PNG phenolic profiles were a consequence of the combination of the environment and the adopted curing technologies between FP and PNG. Indeed, being the exact composition of the *V*. ×*tahitensis* sample from PNG unknown, in principle, we cannot completely exclude also a possible effect of genotype. According to us, the genetic effect, in this case, should be less probable considering that cultivars “Haapape” and “Tahiti,” the two mainly produced cultivars in FP (being “Haapape” the first and “Tahiti” the second most frequently grown cultivars) are also the most widespread cultivars out of FP and in particular in PNG. Further, two phenolic patterns could be distinguished, within the Tahitian main cluster, basically separating the pods from FP batches 1 and 2, and in this case the differences could be mainly a consequence of the different genotypes (Haapape and Tahiti) being the curing method the same and the environmental conditions more similar and uniform. Considering the whole data set, two pods of batch2 were placed within the batch1 cluster. These results confirmed that the pods of FP batch1 have a uniform phenolic profile strongly supporting their belonging to the single cultivar “Haapape.” On the other hand, the FP batch2 was confirmed as a mixture of cultivars and, considering that the 10 pods representing batch2 were randomly selected for the analysis, the most likely scenario is that eight out of ten analyzed pods belong to “Tahiti” and two out of ten belong to “Haapape.”

The OPLS-DA analysis confirmed the results of the cluster analysis, clearly separating the three batches according to their origin (Figure 2), and evidenced that 23 differential metabolites (mainly flavonoids, 12 out of 23 compounds, Table 1 and Figure 2) can provide the same separation power as the cluster analysis. Taken together, these results support the utility of metabolomics in: separating pods based on a different origin and eventually on the different cultivars, even if vines were cultivated in the same area and the pods were processed using the same method; finding the most discriminant metabolites on which eventually base the development of traceability procedures.

Pairwise comparisons were carried out through Volcano plot analysis in order to identify the up- and down-accumulated compounds that can be more interesting in discriminating the different samples. A first comparison was carried out between the two Tahitian samples (Table 2). In this case, 21 compounds were detected to be highly up- or down-accumulated between the two groups (the fold change and the relative abundance of the compounds in the samples is reported in Supplementary Table 3). About half of these compounds were up-regulated in “Haapape” with respect to the mixture. The main differences were among flavonoids (particularly flavonols) and phenolic acids (mainly hydroxycinnamic acids). Amongst flavonols, three compounds were respectively up- and down-regulated in “Haapape”; among cinnamic acids, three and one compounds were respectively up- and down-regulated in the same treatment. These differences can be a consequence of genetic differences between the two cultivars. Indeed, the “Haapape” and “Tahiti” genotypes share common genetic markers, but they differ in their ploidy level, with “Tahiti” being diploid and “Haapape” tetraploid (Lepers-Andrzejewski et al., 2011). This difference can be reflected in a different metabolomics composition of the pods.

By matching the two pairwise comparisons, FP batch1 vs. PNG and FP batch2 vs. PNG, the differential metabolites could be separated in two categories: compounds common between the two comparisons (46 metabolites, Table 3) and compounds exclusive of the single comparisons (7 and 36 metabolites, respectively, Table 4). Among the metabolites reported in Table 3, generally, flavonoids and phenolic acids were the most abundant classes of compounds. Being shared in both pairwise comparisons, we can postulate that these metabolites were independent of the genotype and mainly related to the different origin. This hypothesis is strengthened by the fact that, in both comparisons, the trend of accumulation was always the same for all the compounds reported in Table 3. Furthermore, most of the discriminant metabolites were up-regulated in “Tahitian” batches as compared to the PNG group.

As reported before, the particular cultivar(s) of the PNG sample was/were unknown; nevertheless between batch1 and PNG and between batch2 vs. PNG there were, respectively, 7 and 36 differential molecules. Considering this, the phenolic profile of the PNG sample was more similar to that of batch1 (cultivar “Haapape”), supporting a hypothesis that PNG pods can belong to the cultivar “Haapape” and that the main differences between the two batches are mainly a consequence of the different origin.

Regardless of the cultivar(s) considered, a significantly higher number of differential metabolites was pointed out when geographical origin was adopted as classification criterion under all statistics applied. Similarly, unsupervised hierarchical clustering evidenced that origin was the principal classification parameter. Although discrimination of origin via metabolomics was effective, specific markers could not be pointed out. Indeed, the discrimination potential is related to the actual profile of a wide variety of phenolic compounds, both in terms of their identity and abundance, with flavonoids and hydroxycinnamic acids playing a major role.

Considering our phenolic profiling approach has not been described previously, it becomes difficult to compare our results with markers previously reported in the literature. Nonetheless, most of the markers are aroma-related compounds that are likely more informative regarding vanilla quality rather than its origin. Vanillin is a phenolic aldehyde and one of the most important compounds in the primary extracts from vanilla beans as well as the principal flavor and aroma compound in vanilla. Brunschwig et al. (2016) have reported a significant difference in vanillin content between *V*. ×*tahitensis* grown and processed in PNG and in Tahiti, with a higher concentration in samples from PNG. In this case, vanillin values were significantly different among the three batches by ANOVA, but vanillin was not identified as one of the most differential compounds evidenced by Volcano analysis. This meant that, while a significantly different content of vanillin was present, the fold change value was lower than 2.0 (cut-off value adopted in the Volcano analysis) and that vanillin was not among the compounds mainly influenced by the origin under the present condition of analysis. This difference with respect to Brunschwig et al. (2016) can be a consequence of the different extraction method and of the different analytical technique. On the other hand, it is important to stress out that having several markers is expected to strengthen the discrimination capability of the analytical approach. With this regard, metabolomics followed by multivariate chemometrics is among the approaches gaining most of the popularity in traceability. Important characteristic constituents of *V*. ×*tahitensis* anisyl derivatives (anisyl alcohol, anisaldehyde, methyl anisate, anisyl formate, and anisyl acetate), which were previously detected by HPLC analysis (Brunschwig et al., 2009), were not found as discriminant in this study. However, this can be easily ascribed to their volatility that hampered their ionization efficiency at the electrospray interface. On the other hand, although these compounds are known to play an important role in the vanilla aroma, the whole phenolic profile was wide and differed under both a qualitative and quantitative point of view. With this regard, the latter appeared to be more informative for traceability purposes.

As a conclusion, in the present study, metabolomics focused on the phenolic profile was successfully applied for the first time to *V*. ×*tahitensis* in order to increase our knowledge of vanilla metabolome for traceability purposes. Among the results, the most significant were: (1) the discrimination of the samples based on their origin: PNG samples were clearly different from Tahitian samples; this variation could be mainly related to the different origin (i.e., a combination of pedo-climatic conditions and curing methods adopted in the two countries); (2) the grouping of the Tahitian samples based on the two patterns, which could be explained considering that the first sample corresponded to only one cultivar (“Haapape”) while the second one was a mix of 2 cultivars (“Haapape” and “Tahiti”). These findings evidenced the utility of the metabolomics analysis to detect a high number of discriminating compounds; this possibility, in combination with robust multivariate chemometrics, might open to the possibility to develop a standard procedure for traceability and authentication based on the metabolic profile of the pods. Of course, further studies are necessary to deepen our vision on vanilla metabolome and on the metabolic variations correlated with the cultivars and the origins, in order to detect and then validate selected highly discriminant compounds suitable for authentication. Finally, our deep profiling approach allowed the annotation of unexpected phenolic components, not yet reported, whose presence could be linked to the medicinal properties of vanilla. Polyphenols, such as those identified in this study, significantly benefit human health (Del Rio et al., 2013) and they can deserve further perspective assessments for an up-dated pharmaceutical valuation of vanilla.

## Author contributions

MB designed the study, in cooperation with PR and JF. CB, SL, LB, GS, and JB carried out the plant experiments, contributed to interpretation of data and drafted the manuscript. LL developed the mass spectrometric method, performed statistics and helped to draft the manuscript. MB, PR, and JF draft and critically revise the manuscript. All authors read and approved the final manuscript.

### Conflict of interest statement

The authors declare that the research was conducted in the absence of any commercial or financial relationships that could be construed as a potential conflict of interest.

## Supplementary material

Supplementary Table 1 A full list of the 260 phenolic compounds is provided.

Supplementary Table 2 The compounds, significantly different among the three samples (one-way ANOVA, multiple testing correction, *p*-value 0.05), are reported with the corresponding *P*-value.

Supplementary Table 3 The results of the volcano plot analysis reporting, for each pairwise comparison, the compounds characterized by a fold change higher than 2.
