# Supplementary material for: Phenolic Profiling for Traceability of Vanilla ×tahitensis
Source: Front Plant Sci. 2017 Oct 12;8:1746. doi: 10.3389/fpls.2017.01746 (PMC5644282; doi:10.3389/fpls.2017.01746)
Supplement: Supplementary file 4 [file Image1.PDF]

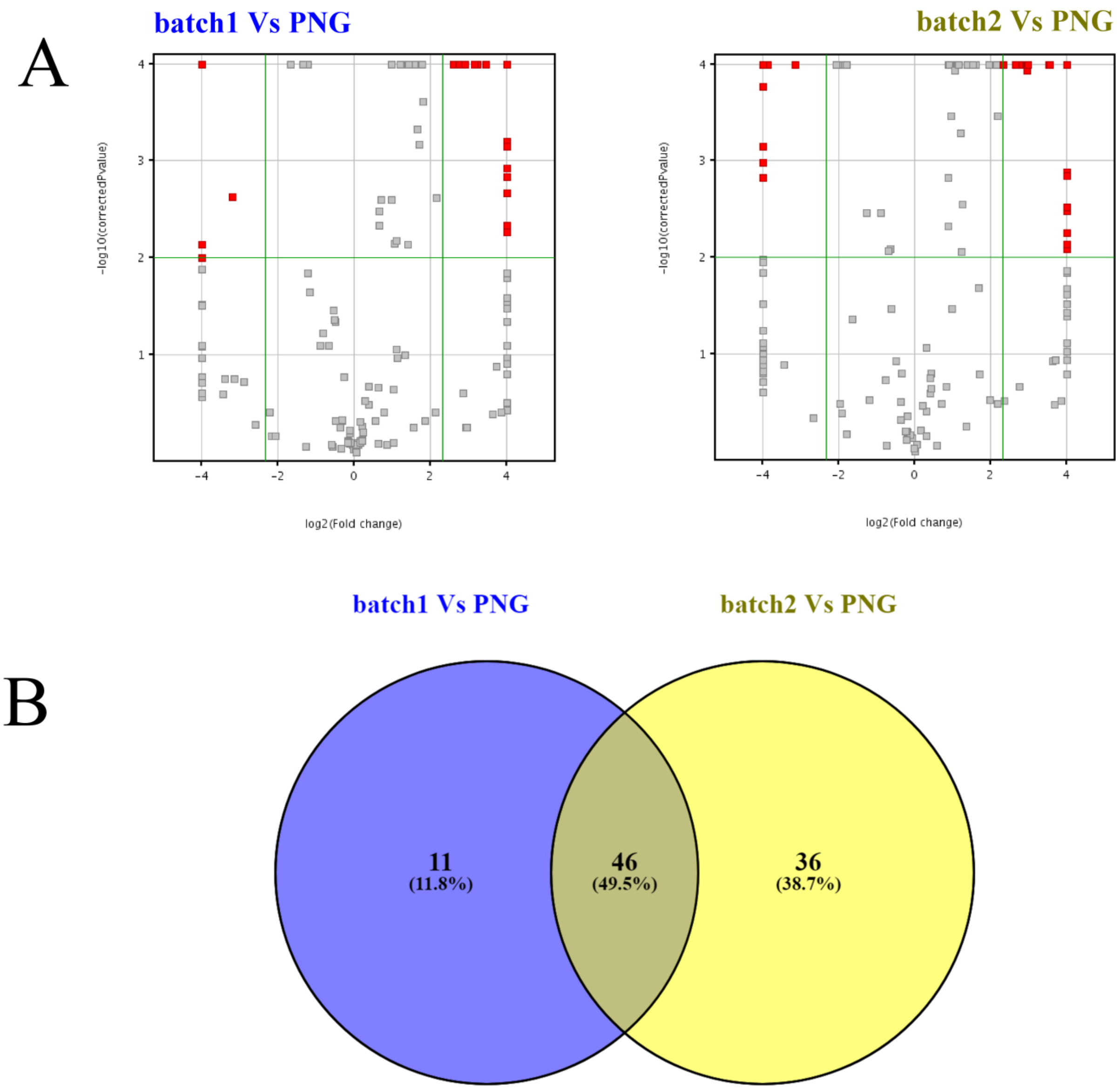

Supplementary Figure 1. 1 A) volcano plot graphic outputs of the comparisons FP batch 1 vs PNG and FP batch 2 vs PNG. The red squares evidence the discriminant compounds between the samples under comparison. The name of the compounds is reported in Table 3 and 4. 1B) Venn diagram, created using the online available tool Venny 2.1.0 – BioingoGP – CSIC, evidencing the number of common (46) and exclusive (11 and 36) compounds between the two pairwise comparisons.
