# Supplementary figures and images for: Phenolic Profiling for Traceability of Vanilla ×tahitensis
Source: Front Plant Sci. 2017 Oct 12;8:1746. doi: 10.3389/fpls.2017.01746 (PMC5644282; doi:10.3389/fpls.2017.01746)

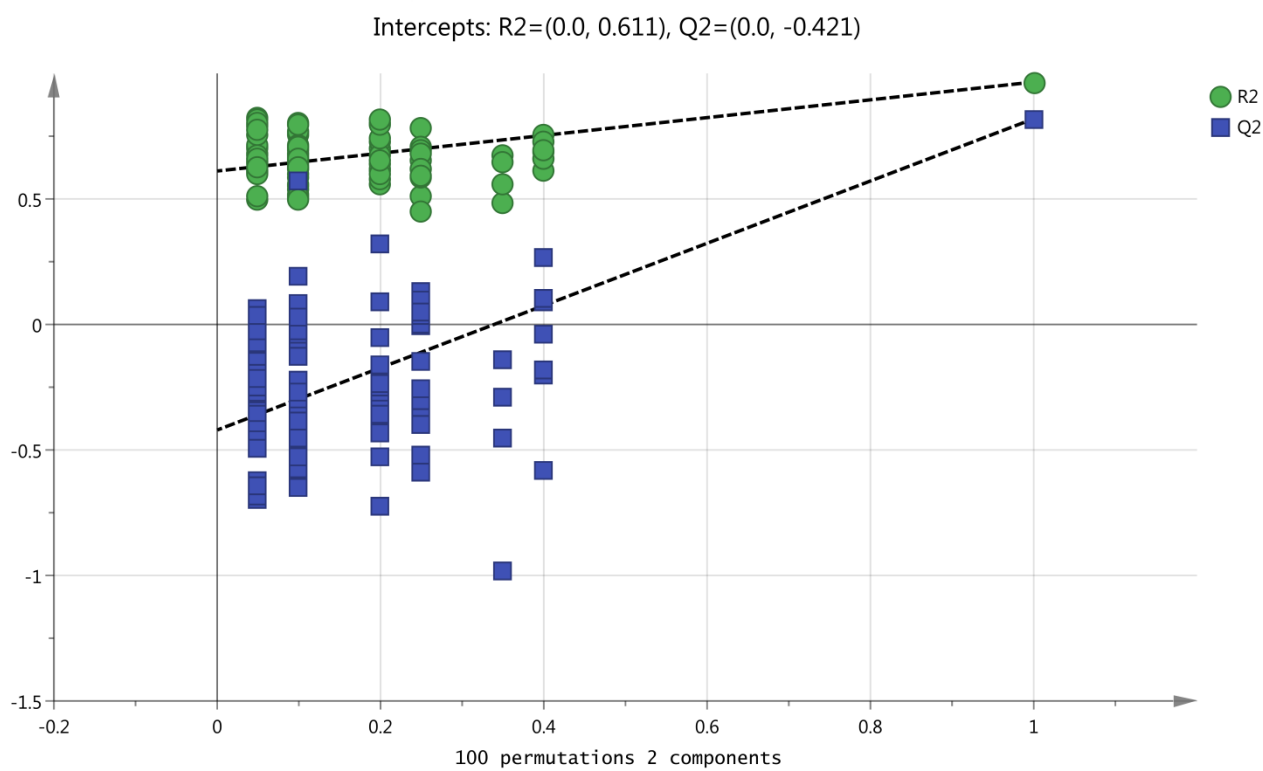

**Supplementary Figure 2.** Permutation test to validate OPLS-DA.

Supplement: Supplementary file 5 [file Image2.PDF]
